# Supplementary material for: Flexible and high-throughput simultaneous profiling of gene expression and chromatin accessibility in single cells
Source: eLife. 2026 Jul 20;15:RP110034. doi: 10.7554/eLife.110034 (PMC13384510; doi:10.7554/eLife.110034)
Supplement: Peer review history

# Editor report: eLife Assessment

**DOI:** 10.7554/eLife.110034.4.sa0

**Authors:** Yamini Dalal

**Affiliations:** National Cancer Institute United States

**Keywords:** Solid, Useful

This study presents a **useful** methodological advance that better enables the simultaneous measurement of gene expression and chromatin accessibility in individual cells. The evidence supporting the improved detection of gene expression is **solid**. The method has the potential to be more broadly impactful if it were expanded to include orthogonal validation strategies. This method will be of interest to those studying transcription and gene regulation.

# Referee report: Reviewer #1 (Public review)

**DOI:** 10.7554/eLife.110034.4.sa1

[Editors' note: this version has been assessed by the Reviewing Editor without further input from the original reviewers. In the latest version, the authors have made textual revisions that note caveats about the quality of the chromatin accessibility data.]

In the manuscript entitled "Flexible and high-throughput simultaneous profiling of gene expression and chromatin accessibility in single cells," Soltys and colleagues present easySHARE-seq, a method described as an improvement upon SHARE-seq for the simultaneous measurement of RNA transcripts and chromatin accessibility.

The authors demonstrate the utility of easySHARE-seq by profiling approximately 20,000 nuclei from the murine liver, successfully annotating cell types and linking cis-regulatory elements to target genes. The authors claim that easySHARE-seq supports longer read lengths potentially enabling better variant discovery or allele-specific signal assessment, though they do not provide direct evidence to support these specific claims.

A key strength of the protocol is enhanced sequencing efficiency, achieved by shortening the Index 1 read from 99 to 17 nucleotides. This reduction does not come at a significant cost to barcode diversity, retaining approximately 3.5 million combinations. Additionally, the approach allows for the sequencing of a sub-library to assess quality prior to final barcoding and sequencing which seems quite clever.

While the increase in RNA transcript recovery is substantial, it appears to come at a cost: there is a notable decrease in ATAC fragments per cell compared to the original SHARE-seq (and other platforms). Likely as a result, the dimensionality reduction (UMAP) shows good resolution for RNA profiles but relatively poor resolution for accessibility profiles. Furthermore, the presented data suggests potential ambient RNA contamination; specifically, the detection of Albumin in HSCs and B cells is likely an artifact of the protocol rather than a biological signal.

Overall, the study is well-presented and represents a promising advance.

# Referee report: Reviewer #2 (Public review)

**DOI:** 10.7554/eLife.110034.4.sa2

Aims:

The authors sought to optimize SHARE-seq, a multimodal single-cell method, to improve the simultaneous profiling of gene expression and chromatin accessibility. Their goal was to enhance barcode design for better sequencing efficiency and cost savings, while improving overall data quality. They then applied their optimized method, easySHARE-seq, to study liver sinusoidal endothelial cells (LSECs) to demonstrate its utility in examining gene regulation and spatial zonation.

Strengths:

The improved barcode design is an advance, increasing the proportion of sequencing reads dedicated to biological information rather than barcode identification. This modification offers practical benefits in terms of sequencing costs and read length, potentially reducing alignment errors. The method also demonstrates improved RNA detection compared to the original SHARE-seq protocol. The biological applications showcase how simultaneous measurement of both modalities enables analyses that would be practically impossible with single-modality approaches, particularly in examining how chromatin states change along developmental or spatial trajectories.

Weaknesses:

There is a notable reduction in chromatin accessibility detection compared to the original SHARE-seq method, likely limiting the use of the method in certain situations.

Overall:

The authors achieve their aim of creating an optimized protocol with improved barcode design and enhanced RNA detection. The method represents a useful advance for specific experimental contexts where the trade-offs are appropriate.

# Author comment: Author response

**DOI:** 10.7554/eLife.110034.4.sa3

**Authors:** Volker Soltys, Moritz A Peters, Dingwen Su, Marek Kucka, Yingguang Frank Chan

**Affiliations:** Friedrich Miescher Laboratory Tübingen Germany; Friedrich Miescher Laboratory Tübingen Germany; Friedrich Miescher Laboratory Tübingen Germany; Max Planck Institute for Biology Tübingen Germany; Groningen Institute for Evolutionary Life Sciences, University of Groningen Groningen Netherlands

The following is the authors’ response to the previous reviews

**Comments from Reviewing Editor:**

I want to share that both reviewers appreciated that this revision has appropriately addressed many of the concerns they raised. However, reviewers concurred that additional wet-lab experiments which validated the findings would have made the work much more impactful; and their concerns about the quality of chromatin accessibility data appear not to be fully resolved. Might I suggest a textual revision that specifically points out these caveats, if you are not able to provide additional data? This would then proceed to VOR without additional need to review. Thanks much for your patience while I assessed the manuscript claims and reviewer opinions.

The changes were very minor (2 sentences in the Discussion and a small section in the Supplementary Notes). It would be great if we could proceed to the VOR stage.
